# Relación de la capacidad funcional y la funcionalidad familiar con la fragilidad en adultos mayores con riesgo cardiovascular en el suroccidente colombiano

**DOI:** 10.7705/biomedica.7473

**Published:** 2024-11-06

**Authors:** Clara Inés Paz, Betsy Mercedes Ledezma, Diana María Rivera, Mabel Lorena Salazar, María Verónica Torres, Franklin René Patiño, Andry Yasmid Mera-Mamián

**Affiliations:** 1 Programa de Fisioterapia, Facultad de Ciencias de la Salud, Universidad del Cauca, Popayán, Colombia Universidad del Cauca Facultad de Ciencias de la Salud Universidad del Cauca Popayán Colombia; 2 Facultad de Fisioterapia, Universidad CES, Medellín, Colombia Universidad CES Facultad de Fisioterapia Universidad CES Medellín Colombia

**Keywords:** adulto mayor, fragilidad, estado funcional, multimorbilidad, envejecimiento saludable, enfermedades cardiovasculares, Aged, frailty, functional status, multimorbidity, healthy aging, cardiovascular diseases

## Abstract

**Introducción.:**

Los cambios del envejecimiento son multidimensionales y multifactoriales, y el síndrome geriátrico de fragilidad es su expresión más problemática y compleja. Este lleva a vulnerabilidad, cambio desproporcionado del estado de salud y declinación funcional, haciéndose necesaria su identificación efectiva y un abordaje integral.

**Objetivo.:**

Describir las características sociodemográficas, clínicas, funcionales y relacionales de la fragilidad en adultos mayores con riesgo cardiovascular en el suroccidente colombiano.

**Materiales y métodos.:**

Se desarrolló un estudio observacional, de tipo transversal, analítico. La población seleccionada fueron adultos mayores inscritos en un programa de riesgo cardiovascular y metabólico en Popayán, Cauca. Mediante un análisis multivariado, se exploró la relación entre fragilidad y algunas variables sociodemográficas, clínicas y funcionales.

**Resultados.:**

Participaron 293 adultos mayores, principalmente mujeres (69,6 %), con edad promedio de 71,23 ± 7,38 años. El 77,1 % se clasificó como independientes en actividades básicas y, el 56,3 %, en actividades instrumentales de la vida diaria; predominó la autonomía en hombres. El 71,1 % de las mujeres y el 43,8 % de los hombres se clasificaron como prefrágiles. En el análisis bivariado, se encontró una relación entre la fragilidad, y las variables sexo, edad, estado civil, nivel educativo, ocupación, perímetro de pantorrilla, capacidad funcional, capacidad instrumental y funcionalidad familiar. El análisis multivariado demostró mayor prevalencia (55 %) de fragilidad o prefragilidad en las mujeres.

**Conclusiones.:**

La mayoría de los participantes se clasificaron como prefrágiles; prevaleció la dependencia y fragilidad en las mujeres, lo que sugiere la necesidad de implementar estrategias de prevención y un abordaje diferencial según el sexo.

## Abstract (en)

**Introduction.:**

The changes associated with aging are multidimensional and multifactorial, with the geriatric syndrome of frailty being its most problematic and complex expression. This syndrome leads to vulnerability, disproportionate changes in health status, and functional decline, making its effective identification and comprehensive management necessary.

**Objective.:**

To describe the sociodemographic, clinical, and functional characteristics of older adults with cardiovascular risk in Southwestern Colombia.

**Materials and methods.:**

This study has an observational, cross-sectional, and analytical design. The selected population included older adults enrolled in a cardiovascular and metabolic risk program in Popayán (Cauca). A multivariate analysis explored the relationship between frailty and certain sociodemographic, clinical, and functional variables.

**Results.:**

A total of 293 older adults participated, primarily women (69.6%), with an average age of 71.23 years. Among them, 77.1% were classified as independent in basic activities and 56.3% in instrumental activities of daily living, with autonomy being more prevalent among men. Additionally, 71.1% of women and 43.8% of men were classified as prefrail. The bivariate analysis identified a relationship between frailty and variables such as sex, age, marital status, educational level, occupation, calf circumference, functional capacity, instrumental capacity, and family functionality. The multivariate analysis showed a higher frailty/prefrailty prevalence (55%) in women.

**Conclusions.:**

Most participants were classified as prefrail; dependency and frailty were more prevalent in women, suggesting the need for preventive strategies and interventions from a gender-differentiated approach.

A nivel mundial, la esperanza de vida -el número promedio de años que se espera viva una persona con el patrón de mortalidad actual- es igual o superior a los 60 años ^1^. Según la Organización Mundial de la Salud (OMS), todos los países están experimentando un crecimiento en cantidad y proporción de adultos mayores. En el panorama mundial, se estima que la población de 60 años o más pasará de 1.000 millones reportados en 2020 a 1.400 millones en 2030, y para el año 2050 se habrá duplicado a 2.100 millones; también, se prevé que el número de octogenarios se triplicará, alcanzando los 426 millones ^2^.

El envejecimiento poblacional en Latinoamérica y el Caribe se percibe desde la segunda mitad del siglo XX y aumenta en forma más rápida que en los países del primer mundo. Este cambio demográfico se considera el más profundo y está ocurriendo en condiciones de gran desigualdad, con el consecuente impacto social, económico y en salud pública. En la década de 1950, los adultos mayores representaban el 5,6 % de la población, en el 2000 aumentaron al 8,3 % y se espera que alcancen el 25 % en el 2050. De esta manera y por primera vez en la historia, la población de más de 60 años será numéricamente mayor que la de 0 a 19 años, con lo que se estima que Latinoamérica y el Caribe tendrán 190 millones de adultos mayores ^3^^,^^4^.

En Colombia, la transición demográfica, marcada por la fecundidad, el número de hijos por familia y la estructura de la población por edad, se ha venido generando de manera lenta. Se hace evidente en el descenso sostenido de la fecundidad, con generaciones cada vez menos numerosas; el cambio demográfico avanza y la diferencia entre la población joven y la de mayor edad es evidente. Además, entre el 2019 y el 2021, la esperanza de vida se redujo cuatro años a causa de la pandemia por COVID-19 ^5^.

Actualmente, se estima que para la próxima década una de cada seis personas tendrá 60 años o más y que, para el 2051, la esperanza de vida será de 83 años: 81 años para los hombres y 85 años para las mujeres ^5^. En el caso de las mujeres, el aumento de la esperanza de vida supone un reto en la urgencia por lograr una redistribución en las actividades del trabajo doméstico y de cuidado no remunerado, y en el mejoramiento de las condiciones para acceder a una pensión y lograr independencia económica. Lo anterior es determinante para el bienestar del adulto mayor ^2^^,^^5^^,^^6^.

Desde la perspectiva biológica, el envejecimiento resulta del acúmulo de una variedad de daños moleculares y celulares a lo largo de la vida, lo que lleva a un descenso gradual de las capacidades físicas y mentales, un mayor riesgo de enfermedad y, en última instancia, la muerte. Estos cambios son multidimensionales y están influenciados fuertemente por la trayectoria de vida, la genética, y el contexto psicosocial y socioeconómico del sujeto ^2^^,^^7^.

La vejez se caracteriza también por la aparición de varios estados de salud complejos que se conocen habitualmente con el nombre de síndromes geriátricos. Por lo general, son consecuencia de múltiples factores subyacentes que incluyen, entre otros, la fragilidad, la incontinencia urinaria, las caídas, los estados delirantes y las úlceras por presión. La expresión más problemática y compleja del envejecimiento de la población es la condición clínica de fragilidad, considerada como un estado de mayor vulnerabilidad debido a la acumulación de daños en múltiples órganos y sistemas que conducen a un cambio desproporcionado en el estado de salud ^6^^,^^8^. Este síndrome también resulta de la interacción entre el proceso de envejecimiento y ciertas enfermedades crónicas, lo que compromete los resultados funcionales de los adultos mayores ^9^.

Se ha evidenciado que el adulto mayor frágil tiene más probabilidades de presentar enfermedad cardiovascular y que esta, a su vez, es un predictor de la aparición de fragilidad, lo que aumenta sustancialmente el riesgo de discapacidad, pérdida de movilidad, caídas y hospitalización, aspectos que afectan la salud, el bienestar y la calidad de vida ^10^. Los datos epidemiológicos disponibles sobre fragilidad se basan principalmente en el fenotipo de fragilidad física; sin embargo, se ha sugerido que la fragilidad debe identificarse mediante un enfoque multidimensional ^11^. Algunas de las herramientas con mayor validez y ampliamente aceptadas para medirla, son el fenotipo de fragilidad de Linda Fried, que aborda la fragilidad como un síndrome, y el índice de fragilidad de Rockwood y Mitnitsky, que la considera un espectro del envejecimiento ^8^^,^^12^.

Dada la heterogeneidad de las manifestaciones clínicas de la fragilidad, a su frecuencia en adultos mayores y a su asociación con enfermedades crónicas -muy prevalentes en el país-, es importante contar con estrategias efectivas para su identificación y con un abordaje que abarque todo el espectro de su gravedad ^8^^,^^13^. El análisis de estos dominios es un insumo importante para favorecer el abordaje integral y multidimensional de los adultos mayores, y para lograr responder a sus necesidades de acompañamiento.

Este estudio tuvo como objetivo principal describir las características sociodemográficas, clínicas, funcionales y relacionales de la fragilidad en adultos mayores con riesgo cardiovascular, en el suroccidente colombiano.

## Materiales y métodos

### Diseño del estudio y población

Se realizó un estudio con enfoque cuantitativo, de tipo observacional y diseño transversal analítico. La población objetivo fueron 1.117 personas de 60 años o más que asistieron al programa de enfermedades crónicas no transmisibles de la Empresa Social del Estado de Popayán (E.S.E. Popayán), en el punto de atención suroccidental de Popayán.

Para calcular el tamaño de la muestra se utilizó la fórmula de estimación de proporciones. Se estableció un nivel de confianza del 95 %, una potencia del 80 %, una precisión del 5 %, una prevalencia preestablecida del 73,9 % y un porcentaje de falta de respuesta del 5 %. La prevalencia preestablecida se tomó a partir de los hallazgos del estudio de Paredes *et al.* sobre "Funcionalidad y factores asociados en el adulto mayor de la ciudad San Juan de Pasto, Colombia" realizado en el 2018 ^14^.

Se determinó que se requerían, como mínimo, 247 participantes. Se hizo un muestreo por conveniencia, según las posibilidades de acceso a la población y la coincidencia con los horarios de prácticas universitarias.

### Recolección de la información

Una vez se contó con el aval de la Universidad del Cauca y la E.S.E. Popayán, se procedió a capacitar profesionales de salud de la institución y estudiantes del programa de fisioterapia de la Universidad del Cauca sobre los objetivos del estudio, los mecanismos de recolección de la información y los primeros auxilios psicológicos. Cada adulto mayor que cumplía con los criterios de selección fue invitado a participar mediante la firma del consentimiento informado, con la cual se procedió a recolectar la información.

### Criterios de selección

Como criterio de inclusión, se consideró la aceptación de los adultos mayores a participar en el estudio mediante la firma del consentimiento informado. Como criterio de exclusión, se consideró algún tipo de afectación física (dolor, fatiga) o mental que impidiera al individuo continuar con la evaluación. Ningún adulto mayor tuvo que ser excluido.

### Variables de estudio

Se consideraron cuatro grupos de variables: sociodemográficas, clínicas, antropométricas y de funcionamiento. Las variables sociodemográficas incluyeron: edad, sexo, estado civil, escolaridad, ocupación, estrato, lugar de residencia, etnia, condición especial, régimen de afiliación en salud y número de hijos. Las variables clínicas fueron: antecedentes personales y familiares, y uso de aditamentos. Las variables antropométricas incluyeron: talla, peso, perímetro abdominal y circunferencia de pantorrilla.

Para determinar la capacidad funcional, se evaluaron las actividades básicas de la vida diaria, mediante la escala de Barthel ^15^, y las actividades instrumentales de la vida diaria, con la escala de Lawton y Brody ^16^. En esta última, se adaptaron los ítems de preparación de comida y lavado de ropa con la opción «no aplica» para los hombres que no ejecutaban la actividad porque no hacía parte de su rol dentro del hogar.

La condición física se evaluó con la batería corta de desempeño físico *(Short Physical Performance Battery,* SPPB) ^17^.

La funcionalidad familiar se evaluó con la escala de percepción de apoyo familiar APGAR *(Adaptation, Partnership, Growth, Affect, and Resolve).* La fragilidad se valoró con los criterios de Linda Fried: pérdida involuntaria de peso, poca energía o agotamiento, lentitud en la movilidad, debilidad muscular y poca actividad física ^18^.

### Análisis estadístico

Las variables cualitativas se describieron mediante frecuencias y porcentajes, y las cuantitativas, mediante medidas de tendencia central y de dispersión, según el tipo de distribución de los datos; la distribución se evaluó con la prueba de Shapiro-Francia.

En el análisis bivariado y mediante la prueba de ji al cuadrado y considerando el criterio de Hosmer-Lemeshow (p < 0,25), se exploró la relación entre fragilidad y variables sociodemográficas como edad, sexo, estado civil, nivel educativo, ocupación, estrato socioeconómico de la vivienda, personas con quienes convive; además, entre fragilidad y las variables antropométricas como perímetro abdominal y de la pantorrilla. En cuanto a las variables del componente de funcionamiento, se exploraron la relación con el uso de aditamentos, la capacidad funcional, la capacidad instrumental y la funcionalidad familiar.

La variable fragilidad fue reagrupada en "no frágil" y "frágil o prefrágil" por la poca frecuencia de sujetos en la categoría «frágil».

Mediante regresiones simples de Poisson, se calcularon las razones de prevalencia (RP) crudas con sus respectivos intervalos de confianza del 95 %; y con una regresión múltiple de Poisson, se calcularon las RP ajustadas. Todos los análisis se realizaron en el programa de acceso libre Jamovi, versión 2.3.28, integrado con RStudio, versión 4.3.2.

### Aspectos éticos

Esta investigación se consideró de riesgo mínimo según la Resolución 8430 de 1993 del Ministerio de Salud de Colombia (19). Todos los procedimientos se realizaron de acuerdo con los estándares éticos de la Declaración de Helsinki de 1964 y sus enmiendas posteriores, y se contó con la aprobación del Comité de Ética para la Investigación Científica de la Universidad del Cauca (acta N° 6.1 - 1.25/32 del 16 de noviembre de 2022).

## Resultados

### Características sociodemográficas generales

Este estudio incluyó 293 adultos mayores que asistieron al programa de enfermedades crónicas no transmisibles de la E.S.E. Popayán. El promedio de edad fue de 71,23 ± 7,38 años. La mayoría de los participantes fueron de sexo femenino (69,6 %), solteros (55,3 %), y su nivel educativo era la primaria (64,3 %). Nueve de cada diez participantes se identificaron como mestizos. Todos se encontraban afiliados al régimen subsidiado. Seis de cada diez adultos mayores reportaron ser residentes de hogares clasificados como asentamientos o de estrato uno. La mayoría informó desempeñarse como amas de casa (48,1 %) y vivir acompañados por familiares (84,3 %) (cuadro 1).

**Cuadro 1:** Características sociodemográficas y laborales

Variable		n	%
Sexo	Masculino	89	30,4
	Femenino	204	69,6
Edad (media ± DE)		71,2	± 7,4
Grupos etarios (años)	60 a 64	56	19,1
	65 a 69	72	24,6
	70 a 74	83	28,3
	75 a 79	42	14,3
	≥ 80	40	13,6
Número de hijos	Sin hijos	31	10,6
	1-3 hijos	138	47,1
	Más de 3 hijos	124	42,3
Etnia	Mestizo	273	93,2
	Indígena	12	4,1
	Afrodescendiente	7	2,4
	Palenquero o de San Basilio	1	0,3
Estado civil	Casado	86	29,4
	Unión libre	45	15,4
	Soltero	162	55,3
Escolaridad	Ninguno	44	15
	Primaria	189	64,5
	Secundaria	47	16
	Técnico/tecnológico	6	2
	Universitario	5	1,7
	Posgrado	2	0,6
Estrato socioeconómico	Asentamiento/Uno	166	56,7
	Dos	76	25,9
	Tres	42	14,3
	Cuatro	9	3,1
Ocupación	Ama de casa	141	48,1
	Empleado	8	2,7
	Trabajador independiente	66	22,5
	Desempleado	78	26.6
Con quien vive	Acompañado	247	84,3
	Solo	46	15,7

DE: desviación estándar

**Figura 1:**
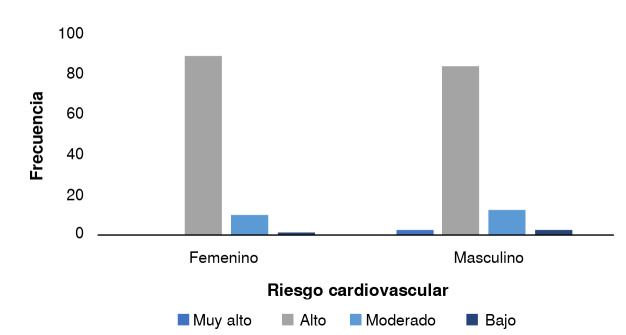
Nivel de riesgo cardiovascular

**Cuadro 2:** Características antropométricas

Variable		Total		Mujeres		Hombres	
		n	%	n	%	n	%
IMC (kg/m^2^)	Mediana (RIC)	26,4 (23,6 - 29 ,8)		26,9 (23,9 - 30,8)		25,2 (22,9 - 28,4)	
Velocidad de la marcha (m/s)		0,8 (0,61 - 0,98)		0,77 (0,57 - 0,92)		0,92 (0,72 - 1,07)	
Fuerza de agarre (kg)		18,2 (13,4 - 23,0)		16 (11,9 - 19,3)		26 (21,1 - 32,3)	
IMC	Delgadez	2	0,7	2	1,0	0	0,0
	Normal	112	38,2	72	35,3	40	44,9
	Sobrepeso	108	36,9	68	33,3	40	44,9
	Obesidad de grado I	51	17,4	43	21,1	8	9,0
	Obesidad de grado II	17	5,8	17	8,3	0	0,0
	Obesidad de grado III	3	1,0	2	1,0	1	1,1
Perímetro abdominal	Normal	74	25,3	40	19,5	34	38,2
	Obesidad abdominal	219	74,7	164	80,4	55	61.8
Perímetro de pantorrilla	> 31 cm	259	88,4	177	86,8	82	92,1
	< 31 cm	34	11,6	27	13,2	7	7,9

IMC: índice de masa corporal; RIC: rango intercuartílico

### Características clínicas y antropométricas según el sexo

El 59,7 % de los participantes reportaron tener diagnóstico solo de hipertensión arterial, el 4,7 % solo de diabetes o prediabetes, y el resto, al menos de dos enfermedades concomitantes, como hipertensión arterial y enfermedad renal crónica. El 86,7 % de los adultos mayores se clasificó con riesgo cardiovascular alto, con resultados similares en hombres y en mujeres (figura 1).

La mediana del índice de masa corporal (IMC) fue de 26,4 kg/m^2^ (RIC: 23,6 - 29,8). En ambos grupos predominaron el peso normal y el sobrepeso, aunque más mujeres (29,4 %) que hombres (9 %) presentaban obesidad de grado I o II. La velocidad de la marcha y la fuerza de agarre fueron mayores en los hombres que en las mujeres. Según el perímetro abdominal, ocho de cada diez mujeres y seis de cada diez hombres, presentaban obesidad abdominal.

Por otra parte, el 11,6 % de los participantes tenía riesgo asociado con discapacidad, sarcopenia y desnutrición, según el perímetro de la pantorrilla (cuadro 2).

### Desempeño físico, capacidad funcional y funcionalidad familiar

El 47,1 % de los adultos mayores, el 53,9 % de las mujeres y el 31,4 % de los hombres tenían un pobre desempeño físico. En cuanto a la funcionalidad familiar, en ambos grupos predominó la categoría «normal». Respecto a la capacidad funcional, la mayoría de los participantes eran autónomos en el desarrollo de las actividades básicas de la vida diaria (77,1 %) y en las actividades instrumentales de la vida diaria (56,3 %). Sin embargo, en estos dos tipos de actividades, más hombres que mujeres eran autónomos. El 71,1 % de las mujeres y el 43,8 % de los hombres se categorizaron como prefrágiles. El 2,9 % de las mujeres se clasificó como frágil, mientras que ningún hombre fue incluido en esta categoría (cuadro 3).

**Cuadro 3:** Desempeño físico, capacidad funcional y funcionalidad familiar

Variables		Total		Mujeres		Hombres	
		n	%	n	%	n	%
Desempeño físico	Normal	155	52,9	94	46,1	61	68,5
	Bajo	138	47,1	110	53,9	28	31,4
Funcionalidad familiar	Normal	226	77,1	152	74,5	74	83,1
	Disfunción leve	36	12,3	31	15,2	5	5,6
	Disfunción moderada	13	4,4	10	4,9	3	3,4
	Disfunción grave	18	6,1	11	5,4	7	7,9
Actividades básicas de la vida diaria	Autónomo	226	77,1	152	74,5	74	83,1
	Dependencia leve	66	22,5	52	25,5	14	15,7
	Dependencia moderada	1	0,3	0	0	1	1,1
Capacidad instrumental	Autónomo	165	56,3	102	50,0	63	70,8
	Dependencia ligera	91	31,1	72	35,3	19	21,3
	Dependencia moderada	23	7,8	19	9,3	4	4,5
	Dependencia grave	13	4,4	11	5,4	2	2,2
	Dependencia total	1	0,3	0	0	1	1,1
Fragilidad	No frágil	103	35,2	53	26,0	50	56,2
	Prefrágil	184	62,8	145	71,1	39	43,8
	Frágil	6	2,0	6	2,9	0	0
Uso de aditamentos	Gafas	133	45,4	93	31,7	40	13,7
	Bastón	20	6,8	9	3,1	11	3,8
	Audífono	9	3,1	7	2,4	2	0,7
	Caminador	2	0,7	0	0	2	0,7
	Muletas	2	0,7	1	0,3	1	0,3
	Ningún aditamento	127	43,3	94	32,1	33	11,3

### Variables relacionadas con la fragilidad

En el análisis bivariado, la fragilidad se relacionó con las variables sexo, edad, estado civil, nivel educativo, ocupación, perímetro de pantorrilla, capacidad funcional (Barthel), capacidad instrumental y funcionalidad familiar (cuadro 3). No se encontró ninguna relación con las variables convivencia, perímetro abdominal, riesgo cardiovascular ni uso de aditamentos (cuadro 4).

**Cuadro 4:** Análisis bivariado de las variables relacionadas con la fragilidad (frágil-prefrágil) en la población de estudio

Variables		No frágil		Frágil o prefrágil		p
		n	%	n	%	
Sexo	Masculino	50	48,5	39	20,5	0,000*
	Femenino	53	51,5	151	79,5	
Grupo etario (años)	60 a 64	20	19,4	36	18,9	0,081*
	65 a 69	32	31,1	40	21,1	
	70 a 74	30	29,1	53	27,9	
	75 a 79	14	13,6	28	14,7	
	≥ 80	7	6,8	33	17,4	
Estado civil	Casado	38	36,9	48	25,3	0,045*
	Unión libre	18	17,5	27	14,2	
	Soltero	47	45,6	115	60,5	
Nivel educativo	Ninguno	11	10,7	33	17,4	0,060*
	Primaria	64	62,1	125	65,8	
	Secundaria o superior	28	27,2	32	16,8	
Ocupación	Ama de casa	41	39,8	100	52,6	0,000*
	Empleado o trabajador independiente	42	40,8	32	16,8	
	Desempleado	20	19,4	58	30,5	
Convivencia	Acompañado	87	84,5	160	84,2	1
	Solo	16	15,5	30	15,8	
Perímetro abdominal	Normal	23	22,3	51	26,8	0,479
	Obesidad abdominal	80	77,7	139	73,2	
Perímetro de pantorrilla	≥ 31 cm	98	95,1	161	84,7	0,014*
	< 31 cm	5	4,9	29	15,3	
Riesgo cardiovascular	Bajo o moderado	13	12,6	23	12,1	1
	Alto o muy alto	90	87,4	167	87,9	
Uso de aditamentos	No	48	46.6	79	41,6	0,481
	Sí	55	53,4	111	58,4	
Capacidad funcional	Autónomo	89	86,4	137	72,1	0,008*
	Dependencia leve o moderada	14	13,6	53	27,9	
Capacidad instrumental	Autónomo	76	73,8	89	46,8	0,000*
	Dependencia leve o superior	27	26,2	101	53,2	
Funcionalidad familiar	Normal	89	86,4	137	72,1	0,008*
	Disfunción leve o Superior	14	13,6	53	27,9	

* Hallazgos estadísticamente significativos (p < 0,25), según el criterio de Hosmer-Lemeshow

En el análisis multivariado, la prevalencia de fragilidad o prefragilidad fue 55 % superior en las mujeres que en los hombres. No se identificó ninguna relación con otras variables, aunque en el análisis crudo sí hubo mayor prevalencia de fragilidad o prefragilidad en los adultos mayores con algún grado de dependencia instrumental, en comparación con aquellos considerados autónomos (cuadro 5).

**Cuadro 5:** Razón de prevalencias de las variables relacionadas con la fragilidad (frágil-prefrágil) en la población de estudio según el análisis multivariado

Variables		RP crudas	IC_95%_	RP ajustadas	IC_95%_
Sexo	Masculino	1		1	
	Femenino	1,69	1,19 - 2,4	1,55	1,01 - 2,40
Estado civil	Casado	1		1	
	Unión libre	1,07	0,67 - 1,72	1,18	0,73 - 1,90
	Soltero	1,27	0,91 - 1,78	1,19	0,84 - 1,72
Ocupación	Ama de casa	1		1	
	Empleado o trabajador independiente	0,61	0,41 - 0,91	0,86	0,55 - 1,36
	Desempleado	1,05	0,76 - 1,45	1,24	0,85 - 1,81
Perímetro de pantorrilla	≥ 31 cm	1		1	
	< 31 cm	1,37	0,92 - 2,04	1,13	0,75 - 1,71
Capacidad funcional	Autónomo	1		1	
	Dependencia leve o moderada	1,30	0,95 - 1,79	1,07	0,76 - 1,51
Capacidad instrumental	Autónomo	1		1	
	Dependencia leve o superior	1,46	1,10 - 1,95	1,23	0,90 - 1,70
Funcionalidad familiar	Normal	1		1	
	Disfunción leve o superior	1,30	0,95 - 1,79	1,18	0,85 - 1,63

RP: razón de prevalencias; DE: desviación estándar

## Discusión

Esta investigación incluyó 293 adultos mayores incluidos en un programa de riesgo cardiovascular. El 69,6 % fueron mujeres, la edad promedio de los participantes fue de 71,23 ± 7,38 años y el estado civil predominante fue la soltería (55,3 %). El 86,7 % fueron clasificados con riesgo cardiovascular alto, con resultados similares en hombres y en mujeres.

Según su capacidad funcional, se consideraron autónomos en actividades básicas de la vida diaria el 77,1 % de los sujetos y, en actividades instrumentales de la vida diaria, el 56,3 %, predominando la autonomía en hombres. El 71,1 % de las mujeres y 43,8 % de los hombres fueron clasificados como prefrágiles y, el 2,9% de las mujeres como frágiles, mientras ningún hombre se clasificó como prefrágil. El análisis multivariado demostró mayor prevalencia (55 %) de fragilidad o prefragilidad en las mujeres.

Con respecto a las características sociodemográficas, el perfil de esta cohorte es similar al encontrado en un estudio de Brasil con 515 adultos mayores en el cual se estudiaron la fragilidad y los factores sociodemográficos asociados, y se reportó que el 70,4 % de la población era de sexo femenino y el 63 % estaba soltero ^20^.

López-Otín *et al.* mencionan que las mujeres pueden tener mayor prevalencia de enfermedades crónicas por factores como la longevidad, ya que en promedio viven más años que los hombres y tienen más probabilidades de desarrollar enfermedades cardiovasculares ^7^. Se sabe que las mujeres suelen tener mayor adhesión a los programas de prevención, pues demuestran mayor cuidado de la salud, conciencia cultural y comunicación efectiva; además, tienen factores de riesgo únicos para enfermedades cardiovasculares, como la menopausia. Esto puede incentivarlas a participar en este tipo de programas ^21^.

En cuanto al estado civil, se ha identificado que es común la soltería, debido a situaciones como la viudez y por falta de oportunidades de pareja a causa de limitaciones sociales, físicas o geográficas, como aislamiento, dificultades de movilidad y falta de acceso a lugares sociales ^22^.

Respecto al nivel educativo y el estrato socioeconómico, más del 70 % de los adultos mayores reportaron contar con escolaridad básica y residir en hogares categorizados como asentamientos o de estrato uno, resultado semejante al reportado en el estudio de Fhon *et al.,* en el cual el nivel educativo predominante fue básica primaria ^20^. En el mismo sentido, en un estudio mexicano se anota que un nivel educativo más elevado está vinculado a mayor entendimiento de los temas de salud y hábitos saludables ^23^. Al respecto, Jiménez *et al.* exponen que estos factores llegan a asociarse con la aparición y el desarrollo de enfermedades crónicas no transmisibles ^24^.

Es importante mencionar que el programa de riesgo cardiovascular de este estudio estuvo disponible para la población vulnerable de la región, donde el acceso a los servicios de salud depende de los subsidios del Estado; por ello, es común encontrar población con estas características educativas y socioeconómicas.

En el presente estudio también se encontró que un gran porcentaje de los participantes se desempeñan en labores domésticas y viven en compañía de su grupo familiar, similar a lo publicado por Fhon *et al.,* en el que más de la mitad de los individuos compartían estas mismas características ^20^. Según la evidencia, esto puede deberse a razones como necesidad de cuidado y vínculos afectivos, ya que con el envejecimiento aumenta la necesidad de algún tipo de asistencia y vivir con miembros de la familia proporciona este apoyo y seguridad emocional. También, algunas personas mayores pueden enfrentar limitaciones físicas o de salud que dificultan las actividades fuera de casa, por lo tanto, se centran más en labores domésticas que pueden desempeñar dentro de su entorno familiar ^25^.

Los resultados obtenidos evidencian que más de la mitad (59 %) de la población reportó antecedentes de hipertensión arterial y otro porcentaje menor la reportó como enfermedad concomitante junto con enfermedades como la diabetes. De manera similar, en el estudio de Díaz *et al.,* la hipertensión arterial se reportó en el 76,8 % de la población estudiada ^26^. Algunos factores físicos determinantes, como las enfermedades crónicas, se han relacionado con la fragilidad del adulto mayor, principalmente la hipertensión arterial que, a su vez, se considera uno de los principales factores de riesgo para desarrollar enfermedad cardiovascular, al favorecer el deterioro de la función física y la fragilidad en general ^27^. Por su parte, la diabetes influye en la función muscular y ósea; algunas de sus complicaciones pueden conducir a la pérdida de masa muscular y a la disminución de la fuerza, lo cual aumenta el riesgo de fragilidad y caídas en los adultos mayores ^28^.

En la población estudiada, se encontró que más del 80 % fue catalogado con riesgo cardiovascular alto, sin distinción de sexo. Esto se explica porque las mujeres y los hombres tienen la misma probabilidad de desarrollar este tipo de padecimientos, pero con diferencias en la manifestación de la enfermedad, los síntomas y el cuadro clínico ^29^.

En cuanto a características antropométricas, el perímetro abdominal ayudó a evidenciar un importante porcentaje de población con obesidad abdominal, resultados que coinciden con la investigación realizada por Díaz *et al.,* quienes describieron esta condición como factor de riesgo para enfermedades cardiovasculares ^26^. Predominaron el peso normal y el sobrepeso en ambos sexos, pero las mujeres obtuvieron un porcentaje mayor en las categorías de obesidad de grado I o II. Al respecto, otros estudios exponen que, en las Américas, el 26 % de los hombres y el 31 % de las mujeres tienen obesidad, y que esta prevalencia puede estar relacionada con sesgos según el sexo, el estado socioeconómico dispar, las tasas de alfabetización disímiles y las deficiencias nutricionales en los primeros años de vida ^23^.

Tanto la velocidad de la marcha como la fuerza de agarre en la población del estudio, fue mayor en los hombres que en las mujeres. En relación con esto, Studenski *et al.* mencionan que factores como la masa muscular y la mayor proporción de fuerza en los hombres podrían contribuir a una marcha más rápida; asimismo, mayor fuerza de agarre puede atribuirse en parte a más masa muscular y densidad ósea ^30^.

El 11,6 % de los participantes se clasificó con riesgo asociado a discapacidad, sarcopenia y desnutrición, según el perímetro de la pantorrilla, lo que eventualmente aumenta la probabilidad de desarrollar un estado de fragilidad debido al deterioro de la función física y la calidad de vida ^31^. Los anteriores son aspectos importantes que se deben considerar ya que, según estudios previos, la relación entre fragilidad y desnutrición parece ser bidireccional. En este sentido, la sarcopenia afecta la capacidad funcional y supone un aumento del riesgo de fragilidad ^32^. Los resultados de este estudio muestran una población no institucionalizada, en su mayoría (77,1 %) autónoma en la ejecución de sus actividades básicas de la vida diaria, similar a lo reportado en un estudio mexicano, en el que el 19,8 % de los adultos mayores encuestados presentaron dependencia leve y el 80,2 % fueron independientes para realizar actividades básicas de la vida diaria ^33^.

Los estudios sobre adultos mayores institucionalizados evidencian una prevalencia elevada de la dependencia funcional. En una investigación realizada en Santander (Colombia), que vinculó 48 participantes, se encontró que el 70 % de los hombres y el 72 % de las mujeres eran dependientes ^34^. Del mismo modo, un estudio de México encontró que el 87,5 % de 32 adultos mayores presentaba algún grado de dependencia funcional ^35^.

La evidencia indica que estas diferencias en los hallazgos entre población institucionalizada y no institucionalizada, podrían explicarse porque la permanencia de los adultos mayores en el hogar ayuda a preservar el grado de autonomía, lo que, a su vez, conlleva ventajas para la salud física, mental, social y emocional ^36^.

Con respecto a las actividades instrumentales de la vida diaria, los resultados de esta investigación demostraron que cuatro de cada diez adultos mayores (43,6 %) son dependientes. En un estudio realizado en el suroccidente colombiano se encontró menores grados de dependencia en las actividades instrumentales de la vida diaria en los adultos mayores no institucionalizados (26,3 %), aunque esta población no tenía un riesgo específico de salud ^14^.

En el presente estudio también se observó que hubo mayor dependencia en las actividades instrumentales de la vida diaria (43,6 %), en comparación con aquellas básicas de la vida diaria (22,8 %). De modo similar, en un estudio de España que incluyó datos de 25.465 personas mayores no institucionalizadas, se concluyó que el 11,1 % de las personas tenían limitación para desempeñar las actividades básicas de la vida diaria, pero existía mayor limitación para las actividades instrumentales (31,9 %) ^37^. En un estudio sobre el envejecimiento cerebral y funcional, realizado en población general en Francia, se concluyó que las actividades instrumentales de la vida diaria son las primeras en perderse porque requieren un funcionamiento cognitivo y físico más complejo ^38^. Por esta razón, estas actividades instrumentales se consideran predictoras de salud y funcionalidad en los adultos mayores ^39^.

Los grados de dependencia en ambos tipos de actividades fueron mayores en las mujeres (25,5 % en las básicas y 50 % en las instrumentales) que en los hombres (16,8 % en las básicas y 29,2 % en las instrumentales). Estos datos son semejantes a los de un estudio en Pasto (Colombia), en el que se encontró mayor dependencia funcional en las mujeres, con 30,9 % para las actividades básicas y para las actividades instrumentales de la vida diaria una media de 6,58 con desviación típica de 1,88 ^40^; y también son similares a los de un estudio de Brasil con adultos mayores no institucionalizados, en el que las mujeres entrevistadas presentaron mayor prevalencia (38,5 %) de dependencia funcional ^41^.

Según el informe de la Organización Panamericana de la Salud (OPS) sobre "Situación de los cuidados a largo plazo en América Latina y el Caribe", las mujeres presentan mayores tasas de dependencia que los hombres, porque tienen mayor esperanza de vida y están más predispuestas a padecer enfermedades crónicas con menor mortalidad, lo cual se refleja en mayor afectación de las actividades instrumentales ^36^.

En esta investigación, el desempeño físico se calificó como bajo o pobre en el 47,1 % de los adultos mayores; estos resultados son similares a los de otro estudio desarrollado en Cali (Colombia), en el cual se reportaron limitaciones de leves a graves en el 44,7 % de la población según el SPPB ^42^, dato importante como factor de riesgo de caídas, afectación de la movilidad y dependencia en la población evaluada.

La participación en actividades cotidianas es relevante para el adecuado desempeño físico, como lo mostró un estudio de la Republica Checa con adultos mayores sin demencia, en el que se obtuvo una media de 10,5 en la escala SPPB, con una desviación estándar de 2,4 (p < 0.0001) ^43^. De igual manera, en un estudio con adultos mayores participantes de un programa de "Gimnasia para la tercera edad", se concluyó que el ejercicio en el adulto mayor mejora las condiciones de salud y la calidad de vida, especialmente en aquellos que llevan mayor tiempo practicándolo (p = 0,014) ^44^.

El sistema familiar de los adultos mayores evaluados en la presente investigación fue percibido como funcional por el 77,1 % de las personas, lo que coincide con un estudio de México, en el que participaron adultos mayores con hipertensión arterial y el 85,4 % manifestó funcionalidad familiar normal ^45^. Resultados similares se reportaron en un estudio de Chile, con adultos mayores sin deterioro cognitivo, en el cual el 78,7 % presentó "buena" funcionalidad familiar ^46^.

Se ha reportado que el establecer relaciones sociales, en especial con familiares, tiene un impacto significativo en la salud física y mental de las personas mayores ^47^^,^^48^. Por el contrario, la funcionalidad familiar inadecuada puede repercutir negativamente en su calidad de vida ^45^.

Con respecto a la fragilidad, este estudio demostró una relación importante con el sexo, siendo las mujeres las más afectadas. Este hallazgo es relevante porque la fragilidad incrementa la vulnerabilidad física, y predispone a un aumento del riesgo de caídas, hospitalización, institucionalización y muerte del adulto mayor ^44^.

En estudios previos se han identificado circunstancias similares y se reporta mayor afectación de las mujeres en las áreas rurales, lo que podría explicarse por factores como las presiones laborales que incluyen las responsabilidades domésticas y de cuidado ^49^. En México, también encontraron que ser mujer era un factor que incrementaba el riesgo de fragilidad, y sugirieron que aspectos como las desigualdades económicas, sociales y en salud pueden marcar estas diferencias ^50^.

Se ha evidenciado que en las mujeres hispanas la fragilidad aumenta entre los 60 y los 80 años (39,5 %), y que perjudica la calidad de vida y la percepción de salud (p < 0,0001) ^51^. En las mujeres frágiles se suelen sumar otros factores que aumentan el riesgo de morir, como el estado civil (divorciadas o separadas), el pobre soporte social y el gran consumo de fármacos ^52^, aspectos que impactan de manera negativa el proceso de envejecimiento y las metas para el logro de una vejez activa y saludable.

La principal limitación del presente estudio estuvo relacionada con el sesgo de selección, debido a que la población fue captada y evaluada según la programación establecida por la institución. Esto implica que los hallazgos no podrían extrapolarse a otras poblaciones, porque los adultos mayores evaluados fueron solo los que pudieron asistir al control médico.

En cuanto a las fortalezas, se destaca el número de personas evaluadas, el uso de instrumentos validados, y la exploración de aspectos como la funcionalidad familiar y otras variables fuera de las sociodemográficas y clínicas. Esto brinda más herramientas a profesionales como los fisioterapeutas que se interesan por abordar a la población desde diferentes aspectos, más que los netamente individuales.

Se concluye que la población estudiada presentó reducción de la variable de capacidad funcional, con mayor afectación en la ejecución de las actividades instrumentales que de las básicas de la vida diaria. Lo anterior debido a que la dependencia funcional evoluciona con el tiempo, lo que puede conducir a una restricción en la participación social.

La mayoría de los adultos mayores del estudio fueron clasificados como prefrágiles y se encontraron diferencias importantes entre hombres y mujeres, ya que ellas presentaron mayor dependencia funcional y fragilidad. Por ello, se considera importante implementar estrategias que procuren disminuir el riesgo de fragilidad, realizando un abordaje diferencial por sexo.

Se evidenció un pobre desempeño físico y un mayor grado de obesidad en las mujeres en comparación con los hombres. Esto demuestra la necesidad de revisar las estrategias educativas para el control de peso implementadas dentro del programa de enfermedades crónicas no trasmisibles de esta institución.

A partir de los hallazgos, se recomienda a la E.S.E. Popayán gestionar la vinculación de los adultos mayores a programas de nutrición para control de peso. También, se sugiere gestionar la articulación institucional con entes territoriales de salud para adoptar medidas de prevención que promuevan la participación de los adultos mayores en programas de actividad física que impacten en su capacidad funcional.
